# Reward and punishment learning in daily life: A replication study

**DOI:** 10.1371/journal.pone.0180753

**Published:** 2017-10-04

**Authors:** Vera E. Heininga, Eeske van Roekel, Marieke Wichers, Albertine J. Oldehinkel

**Affiliations:** 1 Department of Psychiatry, Interdisciplinary Center Psychopathology and Emotion regulation, University of Groningen, University Medical Center Groningen, Groningen, Groningen, The Netherlands; 2 Department of Developmental Psychology, Tilburg university, Tilburg, Noord-Brabant, The Netherlands; Middlesex University, UNITED KINGDOM

## Abstract

Day-to-day experiences are accompanied by feelings of Positive Affect (PA) and Negative Affect (NA). Implicitly, without conscious processing, individuals learn about the reward and punishment value of each context and activity. These associative learning processes, in turn, affect the probability that individuals will re-engage in such activities or seek out that context. So far, implicit learning processes are almost exclusively investigated in controlled laboratory settings and not in daily life. Here we aimed to replicate the first study that investigated implicit learning processes in real life, by means of the Experience Sampling Method (ESM). That is, using an experience-sampling study with 90 time points (three measurements over 30 days), we prospectively measured time spent in social company and amount of physical activity as well as PA and NA in the daily lives of 18-24-year-old young adults (n = 69 with anhedonia, n = 69 without anhedonia). Multilevel analyses showed a punishment learning effect with regard to time spent in company of friends, but not a reward learning effect. Neither reward nor punishment learning effects were found with regard to physical activity. Our study shows promising results for future research on implicit learning processes in daily life, with the proviso of careful consideration of the timescale used. Short-term retrospective ESM design with beeps approximately six hours apart may suffer from mismatch noise that hampers accurate detection of associative learning effects over time.

## Introduction

Emotions are central to everyday life, as they bring flavor to day-to-day experiences. These flavors guide individuals to determine their subsequent actions. Imagine, for example, to experience moments of joy and excitement while being in the company of friends. Without conscious processing, the co-occurrence of this context (i.e., friends) and its positive internal ‘seasoning’ (i.e., feeling joyful and excited) teaches you that spending more time with friends is potentially rewarding. As a result, the probability that one will seek out the company of friends again in the future increases.

The idea that affective experiences motivate actions through implicit associative learning originates from operant conditioning theory. According to Pavlovian or classical conditioning, individuals implicitly (i.e., without conscious processing) learn about the associations between contextual cues and internal cues such as the concurrent levels of affect [1,2]. The rewarding or punishing value of each association, in turn, affects the probability of re-engagement in that activity [3,4]. Although the emotional seasoning of daily life provides an important bias for subsequent motivated action, these implicit learning processes are usually studied in a controlled laboratory setting and not in daily life.

In laboratory settings, implicit learning processes are assessed by experimental tasks, such as the probabilistic reward task [5]; the probabilistic selection task [6]; and the probabilistic reversal learning task [7]. Instead of associations between contextual cues and positive or negative emotions, associations between stimuli and monetary gains or losses are created and manipulated to induce implicit preferences for certain stimuli. For example, when stimulus A is subliminally rewarded with more money than stimulus B (i.e., without conscious processing), individuals are found to develop a preference for A over B [8–10]. Although the laboratory setting for these experiments allows for precise control, the stimuli and the monetary gains and losses are a pale shadow of rewarding or punishing experiences in daily life. Most importantly, it remains unknown how the response biases found in laboratory tasks translate to motivated actions in real-life.

The Experience Sampling Method (ESM) provides an excellent tool to investigate such learning processes because, contrary to the snapshot of an individual’s response bias in laboratory settings, ESM allows investigation of the phenomena over a longer period of time. That is, ESM enables researchers to investigate small but recurrent behavioral and emotional changes across hours, days or weeks, including behavioral consequences of rewarding and punishing experiences. Instead of investigating monetary gains and losses to abstract stimuli in simulation tasks, tracking real-life positive and negative affective experiences to real contextual stimuli and daily activates would enable researchers to investigate response biases in the real world, with findings of high ecological validity. Taken together, the application of ESM to investigate reinforcement learning in real life seems very promising.

Recently, Wichers and colleagues [11] were the first to show that implicit reinforcement learning processes can be studied in real-life by the ESM. They used data of 621 women who were paged at 10 semi-random time points a day, and assessed their emotional state, the extent to which they were physically active, and to what extent they appreciated the company they were in at the time of the beep. Positive Affect (PA) and Negative Affect (NA) experienced in pleasant company affected the degree to which the individuals would seek out pleasant company thereafter in this study. Hence, these findings yielded support for implicit reward and punishment learning processes with regard to engagement in social company. The associations were found both across consecutive beeps and across consecutive days. With regard to physical activity, Wichers and colleagues [11] found similar learning effects, but across a three-day cycle instead of the next beep or day. As a possible explanation, the authors proposed that the body often needs one or two recovery days after sports, during which renewed engagement in physical activity is unlikely.

Three characteristics of the methods and design used by Wichers and colleagues [11] limit the interpretation of their findings. The first concerns the suboptimal measure of social context. Instead of the amount of social interaction or time spent in a certain social context, social company was measured as the extent to which participants liked the company they were in. The level of appraisal of company was found to partially overlap with levels of PA (*r* = .30), which may have inflated the found reward learning effects with regard to social company. The second limitation concerns the timescale. That is, the intervals during the day were relatively short (on average 90 minutes apart), and the activities on beep-level likely often reflect continued rather than newly started activities. Finally, as the authors noted, it would be highly informative to study reward and punishment learning processes in individuals suffering from mental health problems instead of a sample of merely healthy individuals like theirs.

The aim of the current study was to replicate this first Experience Sampling Method (ESM) study into implicit learning processes in real life while overcoming the limitations discussed above. Instead of a measure of social behavior that is interwoven with appraisal, we used more neutral measures of social behavior (i.e., time spent in company; amount of social interaction), and we included specific social contexts (i.e., time spent in company of friends, partner, or family) to explore learning across different types of social contexts. Furthermore, we reduced the probability of spill-over effects due to continuation of activities instead of re-engagement by using beeps that were approximately six hours apart. Finally, we extended the study of Wichers and colleagues [11] by comparing the reward and punishment learning effects in individuals not diagnosed for a mental disorder or in current treatment for a mental disorder to those in individuals suffering from anhedonia. Anhedonia is one of the core symptoms of depression, and defined by the Diagnostic Statistical Manual as a marked loss of pleasure or interest [12], characterized by an impaired ability to pursue, experience, and learn about reward [13–16]. Anhedonic individuals often show reduced reward learning and punishment learning in probabilistic reward tasks [17–20], possibly because they are less able to exploit affective information that guides behavior. Nonetheless, to what extent these altered responses in anhedonic individuals translate to motivated actions in real-life has never been investigated.

Based on the above-described considerations, we hypothesized that levels of PA and NA during social activity would respectively increase and decrease the probability of subsequent engagement in that activity, both within days and across days. For physical activity, we hypothesized that reinforcement learning would follow a 3-day cycle instead of a 1-day or within-day cycle. Furthermore, we hypothesized that individuals with anhedonia would show impaired associative learning rates as compared to individuals without.

## Material and methods

### Sample

**Fig 1:**
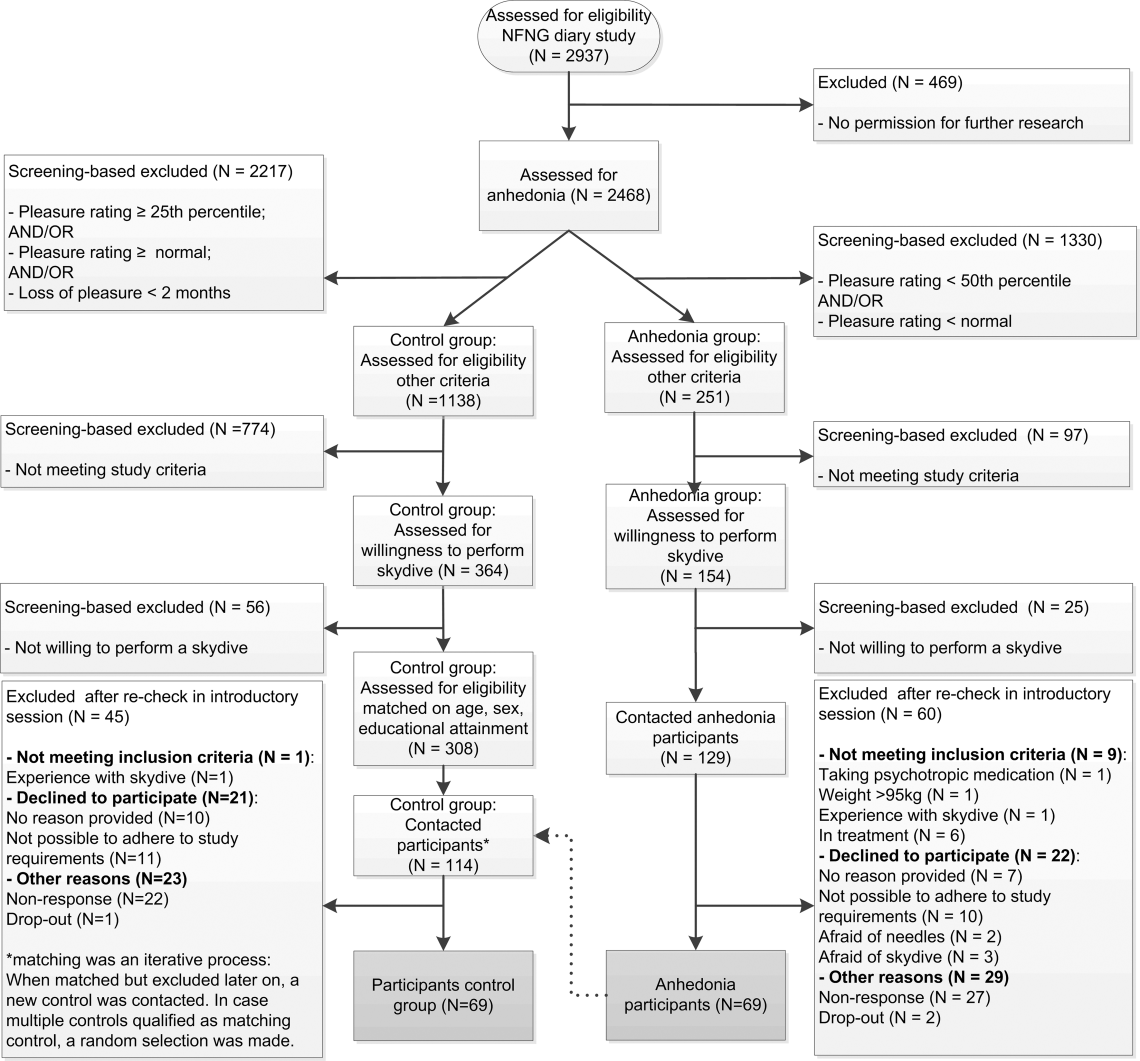
Flowchart of the participant enrollment. Dotted line indicates matching procedure.

The sample is part of the larger No Fun No Gory (NFNG) study: a randomized controlled trial to explore the effects of personalized lifestyle advice and tandem skydives on pleasure levels in anhedonic young adults (see [21]). The NFNG study is registered in the Dutch Clinical Trial Register (NTR5498) and was approved by the Dutch Medical Ethics Committee from the University Medical Center Groningen (no. 2014/508). Participants were selected through an online screening survey among 2937 young adults (M age = 21.4 years, SD = 1.9, 78% women) from the Northern part of the Netherlands. The final sample consisted of a total of 138 ESM participants who met the inclusion criteria and were randomly selected for the ESM-part of the study: 69 with anhedonia, and 69 matched controls. For a more graphic representation of the inclusion and exclusion of participants, please see the flowchart in Fig 1; for more details on the demographics of the anhedonic individuals and matched controls, please see Table 1.

**Table 1:** Demographics of the matched anhedonic and non-anhedonic sample.

	No anhedonia (controls)		Anhedonia	
Men (%)	14	(20)	14	(20)
Mean age in years (SD)	21.51	(1.90)	21.45	(1.96)
Current education (%)				
Tertiary education: University	41	(59)	40	(58)
Tertiary education: Higher vocational	26	(38)	25	(36)
Secondary education	2	(3)	3	(4)
Lower secondary education	0		1	(2)
Compliance rate (%)	83.52	(92.8)	84.33	(93.7)

Age reflects the age in years on the day before the introductory session. N = 69 in both groups. Due to differences in educational systems, current education is grouped based on Fig 1 in Veldman et al. [22]. Highest level of completed education of the three participants without current education were: lower and higher secondary education, and higher vocational education. Three participants reported no current education, and were categorized on the basis of their highest level of education attained (namely lower secondary education; higher secondary; higher vocational). Compliance rate was the average number of assessments filled out of 90, averaged over all participants within that group.

Inclusion criteria for the anhedonia group were 1) persistent anhedonia and 2) willingness to perform a skydive. Persistent anhedonia was defined based on three items from the Domains of Pleasure Scale (DOPS; [23]) assessing (1) the level of pleasure in the past two weeks, (2) whether this level represented a change compared to what is considered normal for this individual, and (3) the duration of the loss of pleasure, if any. In order to be eligible for inclusion of the anhedonia group, a participant was required to report a level of pleasure below the 25th percentile, which was experienced as lower than normal, and had to suffer from this loss of pleasure for at least two months. For the control group, the inclusion criteria were 1) no loss of pleasure (i.e., pleasure rating above the 50th percentile, rated as ‘just as much’, ‘more’ or ‘much more’ compared to their normal levels) and 2) willingness to perform a skydive. Participants with anhedonia who were willing to perform a skydive (answer yes or maybe) did not differ significantly from those who were not willing to do so (*N* = 25) with regard to the severity of anhedonia (t = 1.30, *p* = .13), the level of consummatory pleasure (t = -0.84, *p* = .40), and depressive symptoms (t = 0.30, *p* = .77). Exclusion criteria were: inability to keep an electronic diary three times a day; current professional treatment for psychiatric problems; current use of psychotropic medication; epilepsy; pregnancy; conditions that make it impossible to be attached to the tandem master (e.g., loose prostheses); height of more than two m; weight of more than 95 kg; inability to raise one’s legs 90° (needed for safe landing after tandem skydive); significant visual or hearing impairments; experience with skydiving, bungee jumping, base jumping, or sky jumping; and cardiovascular problems. All criteria were included as questions in the screening survey. After one month of diary study, all participants received a compensation of 75 euros for their participation under the condition that they completed the monthly questionnaires and filled in at least 72 of the 90 momentary assessments (i.e., 80%). In addition, individuals in the anhedonia group needed to provide blood samples before and after the month of diary study to receive the compensation.

### Procedure

Individuals who were eligible for the intervention study and gave permission to be contacted for further research received a detailed information letter and consent form by email. The information letter included information about the study procedures, possible risks, insurance regulations and the possibility to stop the study at any point. After individuals returned a signed consent form, participants were invited for an introductory meeting, and asked to fill in an online questionnaire at home on the preceding day.

During the introductory session, participants’ age (checked in ID), exclusion and inclusion criteria were checked again, study procedures were explained, and participants were instructed on how to fill in the momentary assessments on their smartphone. If still eligible and judged able to adhere to the protocol, participants received an interactive web link in a text message (and e-mails, if desired) at three set time points per day for 30 consecutive days. The web links were sent securely via a web application by RoQua (www.roqua.nl), a system specially designed for routine outcome monitoring in mental health care that meets the highest privacy standards. Except for the nights, the interval between the assessments was always six hours, but the exact times were adapted to the lifestyle of the individual participant (e.g., 9 am – 3 pm– 9 pm). When a diary was not filled in at the scheduled time, participants got a reminder after one hour and another after an hour and a half. The link became unavailable two hours after the first notification. Compliance was monitored daily, and participants were contacted when they missed more than three assessments on three consecutive days, or were close to missing more than 20% of the total number of assessments.

### Measures

#### Affect

Affect was assessed with items similar to those used in the Uncovering the Positive Potential of Emotional Reactivity (UPPER) study [24], which were adapted from previous ecological momentary assessment studies [25,26], and consists of both high arousal and low arousal items. Participants were asked to rate the extent to which they experienced a certain emotion by moving a slider along the continuum of a Visual Analogue Scale (VAS), that was anchored with not at all (left) and very much (right). The location of the slider was converted into a score between 0 and 100. In the afternoon and evening assessments, affect was measured in retrospect (i.e., “Since the last assessment, I have felt [insert adjective emotion]”). To prevent distortion by sleep, morning affect was measured in the moment (i.e., “I feel [insert adjective emotion]”). Positive affect (PA) was calculated by averaging the VAS-scores of the following 10 items: feeling interested, joyful, determined, calm, lively, enthusiastic, relaxed, cheerful, satisfied, and energetic. Negative affect (NA) was calculated by averaging the scores on the following eight items: feeling upset, gloomy, sluggish, anxious, bored, irritated, nervous, and listless. Cronbach’s alpha was .94 for PA and .86 for NA (calculated over all assessments).

#### Social company

Social company was operationalized in five different ways, namely: 1) the amount of social interaction; 2) the amount of time spent in company; 3) the amount of time spent with romantic partner; 4) the amount of time spent with family; and 5) the amount of time spent with friends. The first operationalization was with an adapted item from the Uncovering the Positive Potential of Emotional Reactivity (UPPER) study [24], that read:“[Since the last assessment,] how much did I talk to others?”, followed by VAS anchored with *not at all* (left) and *very much* (right). The other four types of company were assessed by a probe question in which participants were asked to indicate whom they spent time with since the last assessment (answer categories: *Alone / With partner / With family / With friends / With fellow students / With colleagues / With acquaintances / With strangers*), for each option ticked followed by the question: “How much time did I spend with [type of company]?”, which could be answered on a VAS sale ranging from *very little* (left) to *a lot* (right). The VAS-scores were converted into a number between 0 and 100. For the probe question, an unticked box was registered as 0 (i.e., no time spent in that company) and the time registered for “Alone” was inverted to reflect the time that the participant was not alone, and thus in company of others.

#### Physical activity

Physical activity was adapted from previous ecological momentary assessment studies [24,27,28] and assessed by the question: “[Since the last assessment,] I have been physically active:”, followed by a VAS anchored with *not at all* (left) and *very much* (right). The location of the slider was converted into a score between 0 and 100.

### Data analytic plan

Following Wichers and colleagues [11], we used multilevel modelling in which ESM observations (level 1) were clustered within participants (level 2). After we person-centered the level 1 predictors in SPSS version 24.0.0.0 [29], we conducted multilevel regression analyses in MPlus version 7.4 [30]. In order to investigate the effect of implicit reward and punishment learning processes on subsequent activities, social and physical activity at T was operationalized as a function of reinforcement learning at T-1. Because morning affect that was reported in the moment could never co-occurred with activities reported since the last assessment, morning assessments of affect were excluded from the analyses. Reward and punishment learning were operationalized as the interaction effect of, respectively, the level of affect (i.e., PA or NA) at the previous assessment (i.e., T-1) with the level activity at T-1 (i.e., social or physical activity) on the level of that particular activity at T. To explore group differences in these effects, we added individuals’ anhedonic status as a level 2 predictor (control group versus anhedonia group). Mathematically, the random slope model was as follows:

Level 1:

$${Activity}_{ij}=\beta_{0j}+\beta_{1j}({Affect}_{T-1})+\beta_{2j}({Activity}_{T-1})+\beta_{3j}\;({Affect}_{T-1}\times {Activity}_{T-1})+r_{ij}$$

Level 2:

$$\beta_{0j}=\gamma_{00}+\gamma_{01}(anhedonic\;status)+u_{0j}$$

$$\beta_{1j}=\gamma_{10}+\gamma_{11}(anhedonic\;status)+u_{1j}$$

$$\beta_{2j}=\gamma_{20}+\gamma_{21}(anhedonic\;status)+u_{2j}$$

$$\beta_{3j}=\gamma_{30}+\gamma_{31}(anhedonic\;status)+u_{3j}$$

In addition to beep level, and still mirroring the analyses of Wichers et al. [11], the reward- and punishment-driven behavioral activities were also investigated on day level. The mathematical models of the analyses on day level were the same as for beep level except that, before calculating the lagged effects, the level of affect and activity were averaged over days. For the lagged day-level variable of co-occurrence (i.e., interaction term), beep-level lagged variables were used to ensure co-occurrence within the same time frame (e.g., morning PA was multiplied by behavior in the morning).

In total, we tested 26 reinforcement learning effects. On beep level, we tested 6 interaction effects between level of affect at T-1 and level of social or physical activity at T-1 on subsequent behavior at T for PA and 6 interaction effects for NA. We then repeated these 12 beep-level analyses on day-level, making 24 learning effects in total. Additionally, we tested two 3-day-level interaction effects with regard to physical activity, making a total of 26 effects tested.

To maintain a familywise error rate of .05 over all analyses, a Bonferroni-correction of $\alpha^{\prime }=1-{\left(1-\alpha \right)}^{\frac{1}{VeffLi}}$ was applied with *VeffLi* being the ‘effective number’ of independent tests corrected for the correlation amongst the different predictors (in this case: the interaction terms). Using the approach proposed by Li & Ji [31], the effective number of independent variables of our 26 correlated variables was 19. The experiment-wide significance threshold that is required to keep Type I Error Rate at 5% with 19 independent variables is: 0.0027.

## Results

### Descriptive statistics

The study had high compliance rates. On average, both anhedonia and control participants responded to more than 81 of the total 90 beeps (i.e., > 90%). Given the matching procedure, groups did not differ in gender composition, mean age, and educational level (see Table 1).

As shown in Table 2, control participants had spent on average around 15% of their time with their partner, 18% with their family, and 22% with their friends since the last assessment. In total, they had been in company of others 65% of the time, and reported to have been moderately socially interactive on average. With regard to physical activity, participants from the control group reported to be low to moderately active on average. Across the 30 days of study, control group participants reported an average PA level of 64.44 (SD = 9.36), and an average NA level of 14.95 (SD = 7.23).

**Table 2:** Descriptive statistics for key study variables.

	Participants					
	No anhedonia (controls)		Anhedonia		*Difference*	
	Mean	(SD)	Mean	(SD)	*t*_(136)_	*p*
PA	64.44	(9.36)	54.56	(9.67)	-6.10	***
NA	14.95	(7.23)	23.84	(8.98)	6.40	***
Social Interaction	52.19	(11.98)	50.05	(10.92)	-1.10	
Time spent in company	65.47	(11.75)	67.25	(13.68)	0.82	
Time spent with partner	14.97	(17.12)	17.31	(19.87)	0.74	
Time spent with family	17.72	(12.93)	20.68	(13.45)	1.32	
Time spent with Friends	21.87	(12.95)	19.74	(12.11)	-1.00	
Physical Activity	32.46	(10.12)	29.45	(9.41)	-1.81	~

PA = Positive Affect; NA = Negative Affect. Means reflect individuals’ day-level means, aggregated over 30 days within persons. For PA and NA the morning assessment were asked momentary instead of retrospective, and were therefore excluded. N = 69 in both groups. Differences between groups were tested with independent t-tests.

~ p = .05-.10

* p < .05

** p < .01

*** p < .001

Anhedonia was associated with a lower average level of PA, and a higher average level of NA, but not to differences in average social or physical activity (see Table 2).

### Reward and punishment learning

**Fig 2:**
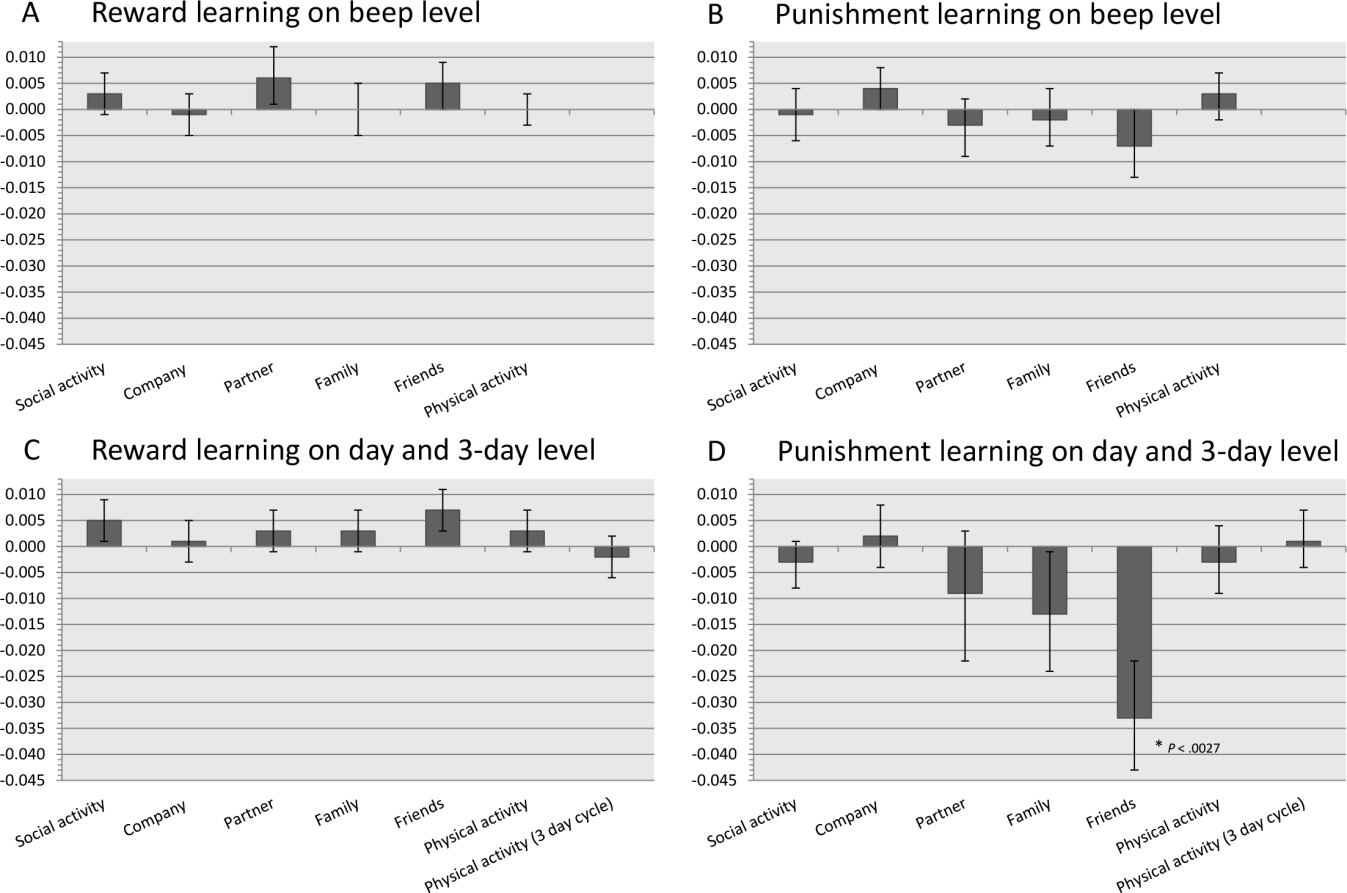
Beta-coefficients for reward and punishment learning at beep level, day level, and 3 day level. Bars represent the beta-coefficients of affect at T-1 by activity at T-1 on activity at T, and reflects the magnitude of these reward punishment learning effects on beep level (Figs 2a & 2b), day and 3 day level (Figs 2c & 2d).

With regard to implicit reward and punishment learning, and as shown in Fig 2 and Tables 3 and 4, the control group did not show modulated behavior as a function of associative learning between level of affect and level of activity on beep- and 3-day-level. That is, none of the reward and punishment learning effects on these levels survived the Bonferroni-corrected statistical level of approximately *p* < .003.

**Table 3:** Estimates of reward learning in individuals without anhedonia and differences of those estimates in individuals with anhedonia.

	Anhedonia status							
	No anhedonia (controls)				Anhedonia			
	Est.	95% CI		*p*	Est.	95% CI		*p*
**BEEP-LEVEL**								
Amount of social Interaction	51.35	[48.29,	54.40]	***	-1.99	[-6.11,	2.12]	
PA_T-1_ on Interaction _T_	0.23	[0.15,	0.32]	***	-0.08	[-0.18,	0.03]	
Social Interaction_T-1_ on Interaction _T_	0.22	[0.18,	0.27]	***	0.01	[-0.06,	0.07]	
(PA_T-1_ × Interaction_T-1_) on Interaction _T_	0.00	[0.00,	0.01]		0.00	[-0.01,	0.00]	
Time spent in Company	63.69	[60.40,	66.99]	***	2.21	[-2.74,	7.17]	
PA_T-1_ on Company _T_	0.19	[0.09,	0.29]	***	0.01	[-0.12,	0.14]	
Company_T-1_ on Company _T_	0.30	[0.26,	0.35]	***	-0.01	[-0.07,	0.06]	
(PA_T-1_ × Company_T-1_) on Company _T_	0.00	[-0.01,	0.00]		0.00	[0.00,	0.01]	
Time spent with Partner	18.09	[13.18,	23.00]	***	3.26	[-4.34,	10.86]	
PA_T-1_ on Partner _T_	0.09	[0.01,	0.16]	*	-0.04	[-0.14,	0.07]	
Partner_T-1_ on Partner _T_	0.48	[0.40,	0.57]	***	0.01	[-0.09,	0.11]	
(PA_T-1_ × Partner_T-1_) on Partner _T_	0.01	[0.00,	0.01]	*	-0.01	[-0.01,	0.00]	*
Time spent with Family	18.82	[15.53,	22.11]	***	2.70	[-2.09,	7.49]	
PA_T-1_ on Family _T_	-0.03	[-0.10,	0.05]		0.00	[-0.11,	0.10]	
Family_T-1_ on family _T_	0.47	[0.41,	0.53]	***	-0.01	[-0.08,	0.07]	
(PA_T-1_ × Family_T-1_) on Family _T_	0.00	[-0.01,	0.01]		0.00	[0.00,	0.01]	
Time spent with Friends	22.64	[19.21,	26.07]	***	-2.14	[-6.70,	2.41]	
PA_T-1_ on Friends _T_	0.25	[0.15,	0.36]	***	-0.10	[-0.24,	0.04]	
Friends_T-1_ on Friends _T_	0.33	[0.29,	0.38]	***	0.01	[-0.06,	0.07]	
(PA_T-1_ × Friends_T-1_) on Friends _T_	0.01	[0.00,	0.01]	*	0.00	[0.00,	0.01]	
Physical Activity	31.19	[28.77,	33.61]	***	-3.78	[-7.13,	-0.43]	*
PA_T-1_ on Physical Activity _T_	0.16	[0.09,	0.24]	***	-0.07	[-0.17,	0.03]	
Physical Activity_T-1_ on Physical Activity _T_	0.07	[0.02,	0.12]	**	0.02	[-0.05,	0.09]	
(PA_T-1_ × Physical Act_T-1_) on Physical Act _T_	0.00	[0.00,	0.00]		0.00	[0.00,	0.00]	
**DAY-LEVEL**								
Amount of social Interaction	51.83	[48.90,	54.75]	***		-2.03	[-5.96,	1.91]	
PA_T-1_ on Interaction _T_	0.25	[0.15,	0.35]	***		-0.05	[-0.17,	0.08]	
Social Interaction_T-1_ on Interaction _T_	0.11	[0.05,	0.16]	***		-0.04	[-0.12,	0.04]	
(PA_T-1_ × Interaction_T-1_) on Interaction _T_	0.01	[0.00,	0.01]	*		0.00	[-0.01,	0.00]	~
Time spent in Company	65.38	[62.51,	68.25]	***		1.67	[-2.69,	6.03]	
PA_T-1_ on Company _T_	0.28	[0.14,	0.41]	***		-0.08	[-0.25,	0.08]	
Company_T-1_ on Company _T_	0.22	[0.17,	0.27]	***		-0.01	[-0.07,	0.06]	
(PA_T-1_ × Company_T-1_) on Company _T_	0.00	[-0.01,	0.01]			0.00	[-0.01,	0.01]	
Time spent with Partner	14.93	[10.89,	18.97]	***		2.50	[-3.70,	8.70]	
PA_T-1_ on Partner _T_	0.18	[0.08,	0.28]	***		-0.08	[-0.21,	0.06]	
Partner_T-1_ on Partner _T_	0.24	[0.18,	0.31]	***		0.01	[-0.09,	0.11]	
(PA_T-1_ × Partner_T-1_) on Partner _T_	0.00	[0.00,	0.01]			0.00	[-0.01,	0.01]	
Time spent with family	17.80	[14.80,	20.80]	***		2.73	[-1.63,	7.09]	
PA_T-1_ on Family _T_	0.07	[-0.04,	0.18]			0.01	[-0.14,	0.16]	
Family_T-1_ on family _T_	0.37	[0.32,	0.43]	***		-0.01	[-0.08,	0.06]	
(PA_T-1_ × Family_T-1_) on Family _T_	0.00	[0.00,	0.01]			0.00	[-0.01,	0.01]	
Time spent with Friends	21.37	[18.37,	24.38]	***		-1.75	[-5.95,	2.45]	
PA_T-1_ on Friends _T_	0.17	[0.05,	0.29]	**		-0.04	[-0.19,	0.11]	
Friends_T-1_ on Friends _T_	0.22	[0.16,	0.27]	***		-0.04	[-0.12,	0.04]	
(PA_T-1_ × Friends_T-1_) on Friends _T_	0.01	[0.00,	0.01]	**		0.00	[-0.01,	0.00]	
Physical Activity	32.20	[29.81,	34.60]	***		-3.01	[-6.25,	0.24]	~
PA_T-1_ on Physical Activity _T_	0.11	[0.03,	0.20]	*		0.01	[-0.10,	0.11]	
Physical Activity _T-1_ on Physical Activity T	0.08	[0.05,	0.12]	***		-0.07	[-0.13,	-0.02]	*
(PA_T-1_ × Physical Act_T-1_) on Physical Act _T_	0.00	[0.00,	0.01]			0.00	[-0.01,	0.00]	
**3-DAY-LEVEL**								
Physical Activity	32.48	[30.10,	34.87]	***		-3.13	[-6.37,	34.87]	~
PA_T-3_ on Physical Activity _T_	0.02	[-0.07,	0.10]			-0.02	[-0.12,	0.08]	
Physical Activity_T-3_ on Physical Activity _T_	-0.02	[-0.06,	0.10]			0.01	[-0.07,	0.09]	
(PA_T-3_ × Physical Act_T-3_) on Physical Act _T_	0.00	[-0.01,	0.00]			0.00	[0.00,	0.01]	

Whereas T-1 on beep-level refers to T minus 1 measurement, on day-level, T-1 refers to T minus the average of 1 day (i.e., average of three beeps on previous day); PA = Positive Affect. On 3-day-level, all estimates all controlled for T-2 and T-1 effects. The number of assessments used on beep-, day-, and 3-day-level were respectively 10739, 3987, and 3710.

Data used for the analyses can be found in S1 and S2 Dataset.

~ p = .05-.10

* p < .05

** p < .01

*** p < .001

**Table 4:** Estimates of punishment learning in individuals without anhedonia and differences of those estimates in individuals with anhedonia.

	Anhedonia status							
	No anhedonia (controls)				Anhedonia			
	Est.	95% CI		*p*	Est.	95% CI		*p*
**BEEP-LEVEL**								
Amount of social Interaction	51.42	[48.39,	54.45]	***	-2.05	[-6.14,	2.03]	
NA_T-1_ on Interaction _T_	-0.14	[-0.25,	-0.03]	*	0.05	[-0.07,	0.18]	
Social Interaction_T-1_ on Interaction _T_	0.24	[0.20,	0.29]	***	0.01	[-0.06,	0.07]	
(NA_T-1_ × Interaction_T-1_) on Interaction _T_	0.00	[-0.01,	0.00]		0.00	[-0.01,	0.01]	
Time spent in Company	63.69	[60.42,	66.96]	***	2.15	[-2.78,	7.08]	
NA_T-1_ on Company _T_	-0.05	[-0.19,	0.10]		-0.06	[-0.23,	0.11]	
Company_T-1_ on Company _T_	0.31	[0.27,	0.36]	***	0.00	[-0.07,	0.06]	
(NA_T-1_ × Company_T-1_) on Company _T_	-0.00	[0.00,	0.01]	*	-0.01	[-0.01,	0.00]	**
Time spent with Partner	18.25	[13.33,	23.17]	***	3.07	[-4.54,	10.67]	
NA_T-1_ on Partner _T_	0.01	[-0.08,	0.11]		-0.06	[-0.18,	0.06]	
Partner_T-1_ on Partner _T_	0.50	[0.41,	0.58]	***	-0.01	[-0.11,	0.09]	
(NA_T-1_ × Partner_T-1_) on Partner _T_	0.00	[-0.01,	0.00]		0.00	[-0.01,	0.01]	
Time spent with Family	18.88	[15.59,	22.16]	***	2.66	[-2.15,	7.46]	
NA_T-1_ on Family _T_	0.00	[-0.09,	0.09]		0.06	[-0.06,	0.18]	
Family_T-1_ on family _T_	0.47	[0.42,	0.53]	***	-0.02	[-0.10,	0.06]	
(NA_T-1_ × Family_T-1_) on Family _T_	0.00	[-0.01,	0.00]		0.00	[-0.01,	0.01]	
Time spent with Friends	22.62	[19.21,	26.03]	***	-2.06	[-6.62,	2.49]	
NA_T-1_ on Friends _T_	-0.16	[-0.28,	-0.04]	**	0.07	[-0.08,	0.22]	
Friends_T-1_ on Friends _T_	0.35	[0.31,	0.39]	***	0.00	[-0.06,	0.06]	
(NA_T-1_ × Friends_T-1_) on Friends _T_	-0.01	[-0.01,	0.00]	*	0.00	[-0.01,	0.01]	
Physical Activity	31.16	[28.74,	33.58]	***	-3.82	[-7.17,	-0.47]	
NA_T-1_ on Physical Activity _T_	-0.08	[-0.17,	0.01]	~	0.04	[-0.07,	0.15]	
Physical Activity_T-1_ on Physical Activity _T_	0.08	[0.03,	0.13]	**	0.02	[-0.05,	0.08]	
(NA_T-1_ × Physical Act_T-1_) on Physical Act _T_	0.00	[0.00,	0.01]		0.00	[-0.01,	0.00]	
**DAY-LEVEL**								
Amount of social Interaction	51.99	[49.11,	54.86]	***	-2.29	[-6.15,	1.57]	
NA_T-1_ on Interaction _T_	-0.18	[-0.30,	-0.05]	**	0.07	[-0.08,	0.21]	
Social Interaction_T-1_ on Interaction _T_	0.13	[0.08,	0.18]	***	-0.04	[-0.11,	0.04]	
(NA_T-1_ × Interaction_T-1_) on Interaction _T_	0.00	[-0.01,	0.00]		0.00	[-0.01,	0.01]	
Time spent in Company	65.48	[62.67,	68.30]	***	2.15	[-2.34,	6.64]	
NA_T-1_ on Company _T_	-0.22	[-0.39,	-0.05]	*	0.06	[-0.14,	0.26]	
Company_T-1_ on Company _T_	0.23	[0.18,	0.28]	***	-0.02	[-0.08,	0.05]	
(NA_T-1_ × Company_T-1_) on Company _T_	0.00	[0.00,	0.01]		0.00	[-0.01,	0.01]	
Time spent with Partner	14.81	[10.74,	18.87]	***	2.11	[-4.07,	8.29]	
NA_T-1_ on Partner _T_	-0.07	[-0.17,	0.03]		-0.02	[-0.15,	0.12]	
Partner_T-1_ on Partner _T_	0.25	[0.18,	0.32]	***	-0.01	[-0.11,	0.09]	
(NA_T-1_ × Partner_T-1_) on Partner _T_	-0.01	[-0.02,	0.00]		-0.01	[-0.02,	0.01]	
Time spent with family	17.56	[14.51,	20.61]	***	2.15	[-2.26,	6.57]	
NA_T-1_ on Family _T_	-0.05	[-0.17,	0.06]		0.04	[-0.12,	0.19]	
Family_T-1_ on family _T_	0.37	[0.32,	0.43]	***	-0.02	[-0.09,	0.05]	
(NA_T-1_ × Family_T-1_) on Family _T_	-0.01	[-0.02,	0.00]	*	0.00	[-0.01,	0.01]	
Time spent with Friends	20.54	[17.40,	23.69]	***	-2.11	[-6.43,	2.21]	
NA_T-1_ on Friends _T_	-0.11	[-0.22,	0.01]	~	-0.05	[-0.19,	0.10]	
Friends_T-1_ on Friends _T_	0.23	[0.19,	0.28]	***	-0.06	[-0.13,	0.01]	
(NA_T-1_ × Friends_T-1_) on Friends _T_	-0.03	[-0.04,	-0.02]	***	0.01	[0.00,	0.02]	
Physical Activity	32.25	[29.88,	34.63]	***	-3.08	[-6.31,	0.15]	
NA_T-1_ on Physical Activity _T_	-0.08	[-0.18,	0.02]		0.01	[-0.11,	0.13]	
Physical Activity _T-1_ on Physical Activity T	0.10	[0.06,	0.14]	***	-0.07	[-0.13,	-0.01]	
(NA_T-1_ × Physical Act_T-1_) on Physical Act _T_	0.00	[-0.01,	0.00]		0.00	[-0.01,	0.01]	
**3-DAY-LEVEL**								
Physical Activity	32.43	[30.03,	34.83]	***	-3.04	[-6.29,	0.21]	
NA_T-3_ on Physical Activity _T_	0.01	[-0.10,	0.11]		0.00	[-0.12,	0.12]	
Physical Activity_T-3_ on Physical Activity _T_	-0.02	[-0.06,	0.03]		0.01	[-0.06,	0.08]	
(NA_T-3_ × Physical Act_T-3_) on Physical Act _T_	0.00	[0.00,	0.01]		0.00	[-0.01,	0.01]	

Whereas T-1 on beep-level refers to T minus 1 measurement, on day-level, T-1 refers to T minus the average of 1 day (i.e., average of three beeps on previous day); NA = Negative Affect. On 3-day-level, all estimates all controlled for T-2 and T-1 effects. The number of assessments used on beep-, day-, and 3-day-level were respectively 10739, 3987, and 3710. Data used for the analyses can be found in S1 and S2 Dataset.

~ p = .05-.10

* p < .05

** p < .01

*** p < .001

On day level, however, a significant punishment learning effect was found with regard to the amount of time spent with friends (*p* < .001; see Table 4). This effect reflects that if, compared to their own average, non-anhedonic individuals had spent more time in company of friends and simultaneously had experienced higher than average levels of NA (i.e., within the same time frame), they tended to spent less time with their friends the next day.

### Reward and punishment learning in anhedonic individuals

Compared to their non-anhedonic counterparts, individuals in the anhedonia group did not differ in reward and punishment learning (see Tables 3 and 4), regardless of the level of investigation.

## Discussion

In this study we aimed to replicate the pioneer finding of Wichers and colleagues [11], while overcoming their limitations. Based on their findings, and basic principles of reinforcement theory [3,4], we expected high levels of PA and NA that occurred during social or physical activities would respectively boost or limit re-engagement in these activities through associative learning processes. Furthermore, based on findings from laboratory research [17–20], we expected that these processes would be impaired in individuals with anhedonia. However, we found neither robust evidence of reward learning or punishment learning processes in non-anhedonic individuals, nor signs of deviations of these abilities in individuals with anhedonia. Of the in total 52 reward and punishment learning effects tested, only one survived Bonferroni correction: participants who had spent more time in company of friends and experienced high levels of NA during the same time interval that day, tended to spent less time with friends the next day.

Perhaps, our measure of associative learning is more ecologically valid than laboratory findings but also harder to capture due to the limited degrees of freedom individuals have to change their behavior in daily life. In real life, humans have often tight schedules of appointments that leave only little room for ad hoc changes based on what is rewarding or punishing and what is not. For example, for those who had a partner, it is likely that they wanted to see him/her more often, but commitments on both sides made that impossible. However, despite possible limited degrees of freedom, we were still able to find a small but significant punishment learning effect with regard to friends. That we found a punishment learning effect, but not a reward learning effect may be due to the inexplicably intertwined nature between friends and PA [32]. That is, experiencing high PA in this specific context is probably the very reason why friends are labelled as such. Experiencing NA while being in company of friends is likely less common [32–34] but of high learning impact, whereas experiencing NA while being in company of family or partner could be more common or socially more accepted and therefore of lower learning impact.

Why we did not find robust evidence of reward and punishment learning effects as found by Wichers and colleagues [11] may be explained by the differences between the two studies. Instead of a *N* = 621 women-sample we used a *N* = 69 mixed-gender sample; instead of appraisal we assessed social activity with multiple, more neutral measures of social behavior (i.e., time spent in company and amount of social interaction instead of appraisal of company); instead of 10 semi-random beeps on five consecutive days, we used two assessments per day for the co-occurrence of affect and behavior for 30 consecutive days. Although social and physical activity may slightly differ between sexes (see Fig 1 in [35]; [36]), it is highly unlikely that these factors would cause disappearance of most reward and punishment learning effects when using a more mixed sample instead of a mere women sample.

Given the small effect sizes, high sample size, and that without correction for multiple testing the risk of a false positive can capitalize to over 50% when exploring fifteen or more models, we cannot exclude that at least some of the findings reported by Wichers and colleagues are falsely flagged. However, given reinforcement theory and the supportive evidence from laboratory studies [17–20], it is at least equally plausible that we did not accurately capture the implicit associative learning processes. We mathematically modelled interaction terms as the multiplicative of affect and behavior reported since the last assessment of approximately six hours and, given that the associative learning process requires simultaneous presence of two stimuli [1,3,4], this time span may have been too large to accurately capture the co-occurrence between social or physical behavior and affect. Participants could have engaged in a hodge-podge of activities since the last assessment (or multiple engagement of these activities separately) for which all an interaction term was calculated with the levels of PA or NA whereas, in reality, perhaps only one of those activities was actually accompanied by heightened PA or NA. Still, if there was a co-occurrence between behavior and affect, we would have been captured it. Taken together, our assessment of joint appearance may thus have been less accurate but not unreliable.

Although inaccuracy cannot explain our robust lack of findings, a lack of power can. That is, because affect was measured differently in the mornings (i.e., momentary instead of retrospectively), the remaining two time points may have left us unable to detect any reinforcement learning effects in daily life–especially with regard to the specific types of social behavior (i.e., family, friends, or partner). That is, the amount of engagement in the specific types of social behavior were conditional questions and recoded to reflect zero if missing. Post-hoc cross tabulations of the number of afternoon and evening assessments showed that 38% and 40% of all assessments included time spent with family and/or with friends, and less than 24% included time spent with partner (i.e., time spent was non-zero, ranging from very little to all time spent in this type of social context since the last assessment). In sum, the two instead of three assessments a day for which the co-occurrence of affect and behavior could be calculated, in combination with the lower engagement in the specific types of social behavior, may not have provided enough power to detect effects of previous associative learning–especially for those behaviors that were asked conditionally. To properly study reward and punishment learning in real life, future ESM studies should thus benefit from a priori power analyses for each of the behaviors under study but also of its effective time points of co-occurrence.

Another possibility is that our operationalization of implicit learning characterizes a different concept than the implicit learning concept studied in controlled labs. We tried to deduce implicit learning processes from the predictive power of affect experienced in co-occurrence with real-life activities, as assessed by means of experience-sampling methods. In controlled labs, stimuli and rewards that mimic real-life have been found to associate subconsciously, and reward and punishment learning have been studied by manipulating the rewarding and punishing value (e.g., monetary gain or loss) of a stimulus (e.g., symbol) during a reward learning task. Contrary to controlled laboratory experiments, we did not manipulate the rewarding or punishing value of the stimuli under study, but tested whether the level of behavior (social and physical activities) interacted with the concurrently assessed level of positive or negative affect to predict future behavior. Possibly the learning processes captured in the flow of daily life differ from those captured in the lab, and implicit learning measured by experience sampling constitutes a different concept or aspect of implicit learning from that studied in controlled labs.

Given the limitations described above, it is encouraging that we were still able to find suggestive evidence for the learning effect in daily life. Trends of the interaction effects were all but one in the right direction, and without Bonferroni-correction several reached significance. Although the effects with a value above the Bonferroni-corrected *p* of .003 should be interpreted with caution, this pattern of findings may signal potential for the use of ESM to investigate motivated action in daily life. In addition to study designs with semi-random beeps and in-the-moment assessments of affect and behavior, short-term retrospective studies with fixed beeps hours apart may be equally ecologically valid and reliable source for investigating daily reinforcement learning. Future short-term retrospective ESM studies on reward and punishment learning would benefit from creating more discriminatory power by asking participants for the exact timing of affect and behavior or its co-occurrence.

We predicted social behavior using a within-subject affective decision-making theory stating that, when stimuli X and Y are presented together, X is subconsciously associated to Y, and the X-Y association influences subsequent decision-making. In addition to Pavlovian theory, re-engagement in social behavior could also be explained by the evolutionary game theory. According to the game theory, reward and punishment learning capacities evolved as means to cooperate with others [37]. Game theory is built on the assumption that individuals act rationally, that is, cooperate or defect in interaction with others in line with their own interests. Whether cooperation is in one’s interest is partly based on learning and experience. In the context of social dilemmas, reward and punishment learning may have important implications for the viability of behavior and the decision to re-engage in social activities or contexts (see, for example [36–38]).

With regard to the differences we found in time expenditure of individuals with anhedonia, some other trends may also be of interest for future replication studies. Individuals in the anhedonia group reported diminished PA and higher NA, appeared somewhat less physically active but showed no differences in social activity. Previously, the frequency of engagement in social and physical behaviors was related to mild depression [39] and the experience of fewer positive events to anhedonia in MDD [40]. Although some studies also reported no differences in the daily activities of depressed individuals nor in the amount of time spent alone [41], the majority of findings point toward a difference in perception of social activities (i.e., the extent to which these activities are rated as positive or enjoyable) rather than a difference in the absolute number of social activities undertaken. That we found anhedonia unrelated to time spent in social context, in combination with an increased punishment learning curve with regard to spending time in company, suggests that anhedonia may not make individuals less socially active per se, but rather more prone to social withdrawal after experiencing negative emotions in company of others. Given that these findings should be interpreted with caution, however, replication is warranted.

### Strengths and limitations

The present study has many strengths. First, the study uses ESM to study reinforcement learning, a study method that has higher ecological validity than laboratory tasks. Second, the study was the first to explore ESM reinforcement learning effects in a clinical sample. Third, for an ESM study, the study has a long duration compared to other ESM studies. Fourth, we had multiple measures of social company available, all of which slightly differed in their degree of effort allocation (e.g., social interaction versus being not alone) and in their specificity of company (e.g., being not alone versus being with friends).

Despite its many strengths, the findings have also some limitations with regard to their generalizability. First, the sample comprises predominantly highly educated young adults of which only 20% is male. Although never examined in daily life there may well be sex differences in daily reward and punishment learning (see, for example [37]). If there are sex differences in reward and punishment learning in daily life, our results are thus probably more generalizable to women than to men. Second, because affect was measured differently in the mornings (i.e., momentary instead of retrospectively), the modelled interaction effects did not include the associative learning processes that may have occurred during evenings, nights, or early mornings. Third, physical activity was assessed as the overall amount of subjective activity, whereas more detailed information on its intensity and duration may have been more informative. Research shows that PA increases after engaging in physical activities of low-to-moderate intensity, but only if the physical activity lasts for at least 30 minutes [42,43]. Ideally, we would have used an objective assessments of physical activity, for example, via an actigraph accelerometer or smartphone auto-registration [44].

## Conclusion

Our study shows promising results that reward and punishment learning processes can be observed in real life. Careful consideration of power and timescale, however, is key. Short-term retrospective ESM design with beeps approximately six hours apart may suffer from mismatch noise that hampers accurate detection of associative learning effects over time. Future research is needed to determine to what extent the time frame of retrospective designs could be used without information on co-occurrence, and whether learning processes can be captured over more than six hours when individuals report on the co-occurrence of behavior and affect.

## Supporting information

S1 Dataset Data used for beep-level analyses. (SAV)

S2 Dataset Data used for day-level analyses. (SAV)
